# Complete sequence analysis of the mitochondrial genome of *Auriculastra duplicata* (Mollusca, Gastropoda, Ellobiidae)

**DOI:** 10.1080/23802359.2017.1398614

**Published:** 2017-11-08

**Authors:** Chang Ho Yi, Keun-Yong Kim, Tae Won Jung, In-Young Cho, Il Hun Kim, Soon-Sang Hong, Sung-Jin Hwang, Moongeun Yoon, Won Kim, Donguk Han, Min-Seop Kim

**Affiliations:** a National Marine Biodiversity Institute of Korea, Janghang-eup, Republic of Korea;; b School of Biological Sciences, College of Natural Sciences, Seoul National University, Seoul, Republic of Korea;; c Korea Maritime and Ocean University, Busan, Republic of Korea;; d Museum für Naturkunde Berlin, Berlin, Germany;; e Department of Eco-Biological Science, Woosuk University, Wanju, Republic of Korea

**Keywords:** *Auriculastra duplicata*, Ellobiidae, mitochondrial genome

## Abstract

The mitochondrial genome of the gastropod *Auriculastra duplicata* was completely sequenced. It was 13,920 bp in length and comprised 37 genes; two *rrn* genes and 22 *trn* genes. Phylogenetic analyses based on the concatenated protein-coding genes depicted the polyphyly of all species belonging to the family Ellobiidae; however, monophyly was observed among all species belonging to the subfamily Ellobiinae, in which *A. duplicata* clustered consistently with *Auriculinella bidentata*.

*Auriculastra duplicata* (Pfeffer 1854) is a pulmonate gastropod inhabiting the intertidal to terrestrial regions of the estuaries and coasts. *Auriculastra duplicata* was reported for the first time from a salt marsh along the coasts of the Yellow Sea of South Korea in 2015 (Lee and Lee 2015), wherein the marine environments have been affected by pollution, reclamation, and coastal development during the last few decades.

*Auriculastra duplicata* investigated in this study was sampled from Ganghwa-gun, South Korea in 2015. Its voucher specimen (MABIK#MO00163733) was deposited in the National Marine Biodiversity Institute of Korea (MABIK) (Seocheon, South Korea).

Two independent and overlapping PCR runs were conducted to amplify the complete mitogenomic sequence of *A. duplicata*. The PCR product was purified and directly sequenced using the primer walking method. Its complete mitogenome sequence was deposited in GenBank (http://www.ncbi.nlm.nih.gov/) with accession no. MF962898.

All mitogenomic sequences of the family Ellobiidae and its related species were retrieved from GenBank and were aligned with *A. duplicata* mitogenomic sequence obtained in this study. Their respective nucleotide sequences of protein-coding genes were aligned by codons after taking into account the amino acid sequences and the nucleotide matrix of the ten protein-coding genes after excluding three genes (i.e. *nad6*, *nad4L*, and *atp8*) was partitioned into first and second positions of the codon triplets. Maximum-likelihood (ML) and Bayesian inference (BI) analyses were performed using RAxML 7.0.4 (Stamatakis 2006) and MrBayes 3.1.2 (Ronquist and Huelsenbeck 2003) software (Figure 1).

**Figure 1.**
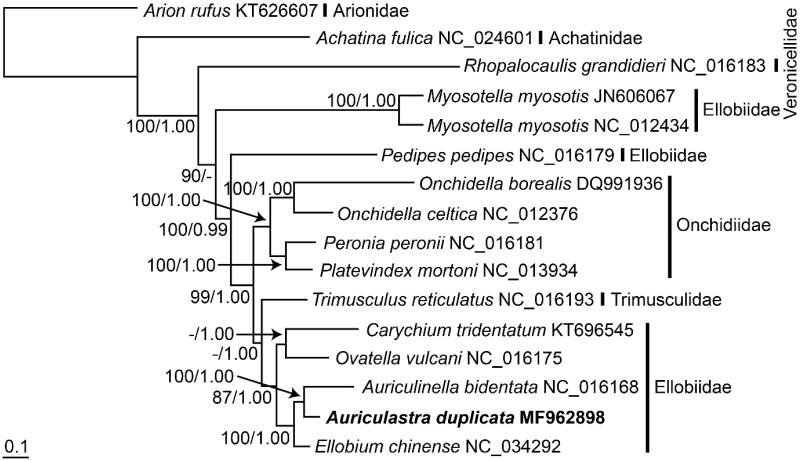
Maximum-likelihood (ML) tree inferred from the mitogenomic sequences of the species belonging to the family Ellobiidae and its related species. The sequence matrix used in the phylogenetic analyses consisted of unambiguously aligned regions of the first and second codon positions of the protein-coding genes. Bootstrap values above 50% in the ML analysis and posterior probabilities above 0.90 in the Bayesian inference analysis are indicated at each node. *Auriculastra duplicata* investigated in this study is shown in bold.

The mitogenomic sequence of *A. duplicata* analyzed in this study was a circular molecule of 13,920 bp in length and very similar to those of *E. chinense* (NC_034292; 13,979 bp), *Carychium tridentatum* (KT696545; 13,908 bp), and *Auriculinella bidentata* (NC_016168; 14,135 bp); all of which belong to the same family (White et al. 2011; Jun et al. 2016; Romero et al. 2016).

A total of 37 genes were identified in *A. duplicata*, which comprised 13 protein-coding genes; two *rrn* genes, and 22 *trn* genes. Its gene content and arrangement were very similar to other species belonging to the family Ellobiidae (White et al. 2011; Romero et al. 2016).

The phylogenetic tree of the species belonging to the clade Eupulmonata was reconstructed with the mitogenomic sequence matrix retrieved from concatenated protein-coding genes. The phylogenetic tree depicted the polyphyletic relationship of the seven species belonging to the family Ellobiidae with respect to two outgroups. *Myosotella myosotis* and *Pedipes pedipes* were separated from the other five species (*C. tridentatum*, *Ovatella vulcani*, *A. bidentate*, *A. duplicata,* and *E. chinense*) that formed the monophyletic group with strong statistical supports in both ML and BI trees. Instead, the five species clustered with the species belonging to the families Onchidiidae and Trimusculidae with strong statistical supports (99% bootstrap value and 1.00 posterior probability). They were further separated, and three species (*A. bidentata*, *A. duplicata*, and *E. chinense*) belonging to the subfamily Ellobiinae (Romero et al. 2016) clustered together with strong statistical supports in both ML and BI trees. *Auriculastra duplicata* analyzed in this study shared the closest phylogenetic relationship with *A. bidentata.*
